# Therapeutic DNA Vaccine Induces Broad T Cell Responses in the Gut and Sustained Protection from Viral Rebound and AIDS in SIV-Infected Rhesus Macaques

**DOI:** 10.1371/journal.pone.0033715

**Published:** 2012-03-19

**Authors:** Deborah Heydenburg Fuller, Premeela Rajakumar, Jenny W. Che, Amithi Narendran, Julia Nyaundi, Heather Michael, Eric J. Yager, Cristy Stagnar, Brendon Wahlberg, Rachel Taber, Joel R. Haynes, Fiona C. Cook, Peter Ertl, John Tite, Angela M. Amedee, Michael Murphey-Corb

**Affiliations:** 1 Department of Microbiology and Molecular Genetics, University of Pittsburgh, Pittsburgh, Pennsylvania, United States of America; 2 Albany Medical College, Albany, New York, United States of America; 3 PowderJect Vaccines, Inc., Madison, Wisconsin, United States of America; 4 GlaxoSmithKline, Stevenage, United Kingdom; 5 Louisiana State University Health Sciences Center, New Orleans, Louisiana, United States of America; Lady Davis Institute for Medical Research, Canada

## Abstract

Immunotherapies that induce durable immune control of chronic HIV infection may eliminate the need for life-long dependence on drugs. We investigated a DNA vaccine formulated with a novel genetic adjuvant that stimulates immune responses in the blood and gut for the ability to improve therapy in rhesus macaques chronically infected with SIV. Using the SIV-macaque model for AIDS, we show that epidermal co-delivery of plasmids expressing SIV Gag, RT, Nef and Env, and the mucosal adjuvant, heat-labile *E. coli* enterotoxin (LT), during antiretroviral therapy (ART) induced a substantial 2–4-log fold reduction in mean virus burden in both the gut and blood when compared to unvaccinated controls and provided durable protection from viral rebound and disease progression after the drug was discontinued. This effect was associated with significant increases in IFN-γ T cell responses in both the blood and gut and SIV-specific CD8+ T cells with dual TNF-α and cytolytic effector functions in the blood. Importantly, a broader specificity in the T cell response seen in the gut, but not the blood, significantly correlated with a reduction in virus production in mucosal tissues and a lower virus burden in plasma. We conclude that immunizing with vaccines that induce immune responses in mucosal gut tissue could reduce residual viral reservoirs during drug therapy and improve long-term treatment of HIV infection in humans.

## Introduction

Although antiretroviral drugs exert considerable control of HIV infection, they do not eliminate virus in the tissues or fully restore virus-specific immunity and interruption of therapy usually results in viral rebound [1], [2], [3]. Because CD4+ and CD8+ T cells play a critical role in controlling chronic HIV infection [4], the goal of therapeutic vaccination is to stimulate these responses during antiretroviral drug therapy (ART) and induce durable immune control of the virus even after ART is discontinued. In this setting, an effective therapeutic vaccine would free HIV-1 infected persons from the complexities of continuous drug dosing, reduce exposure to drugs and associated toxicities, and reduce the potential to transmit virus.

Studies employing therapeutic immunization with peptide-pulsed dendritic cells or PBMC [5], [6], viral vectored vaccines [7], or DNA vaccines [8], [9] support this concept in that therapeutic vaccination with these approaches has been shown to enhance virus-specific T cell responses, reduce viral set-point after withdrawing drugs, and slow or prevent disease progression in SIV-infected macaques. Some of these approaches also had some immunological impact and virological benefit in chronically HIV-1 infected patients [10], [11], [12]. However, durable protection from viral rebound after withdrawing ART has been more difficult to achieve and the immune responses required for long term protection from viral rebound and progression to AIDS after stopping HAART are not yet defined. The gut associated lymphoid tissue (GALT) is a primary reservoir of persistent virus that is inadequately controlled by HAART [13], [14]. Therapeutic vaccines that stimulate mucosal immune responses in the gut could provide a means to more effectively target and control viral replication in this reservoir but to date the impact of a therapeutic vaccine on virus in the gut or other tissue reservoirs has not been investigated.

DNA vaccines are potent inducers of virus-specific T cell responses [15], and studies have shown that prophylactic DNA vaccines, administered either alone or with recombinant viral vaccines, can provide protection against challenges with avirulent and homologous, pathogenic AIDS viruses [16], [17], [18], [19], [20], [21]. Our laboratory previously showed significant prophylactic protection in the SIV model using particle mediated epidermal delivery (PMED; gene gun) of a DNA vaccine [22]. In that study, PMED DNA immunization induced SIV-specific antibody and CD8+ T cell responses in the blood and also in the gut mucosa of macaques. Importantly, despite modest responses in the blood, the vaccine provided complete protection from a disseminated infection in 4 of 7 animals following a high dose rectal challenge with SIV/DeltaB670, a primary isolate that is neutralization resistant [23] and heterologous to the vaccine. Protection following a mucosal challenge in that study strongly indicated that the mucosal responses induced by the PMED DNA vaccine likely played a key role in preventing viral dissemination. In the present study, we investigate the feasibility of administering a therapeutic PMED DNA vaccine formulated with a mucosal adjuvant during ART as a means to augment mucosal T cell responses and target the persistent viral reservoir in the GALT.

Here we show that immunization of chronically SIV-infected macaques with a therapeutic SIV DNA vaccine, together with a plasmid expressing the potent mucosal adjuvant, the heat-labile enterotoxin from *E. coli* (LT), during antiretroviral therapy (ART) increased systemic and mucosal T cell responses, significantly reduced virus burden in the blood and gut tissues, and provided durable protection from viral rebound and progression to disease after stopping ART in the majority of animals that responded well to antiretroviral drugs. These studies also show, for the first time, that a mucosally targeted therapeutic vaccine can induce responses unique to the gut that correlate with therapeutic efficacy.

## Results

### Study design

**Figure 1:**
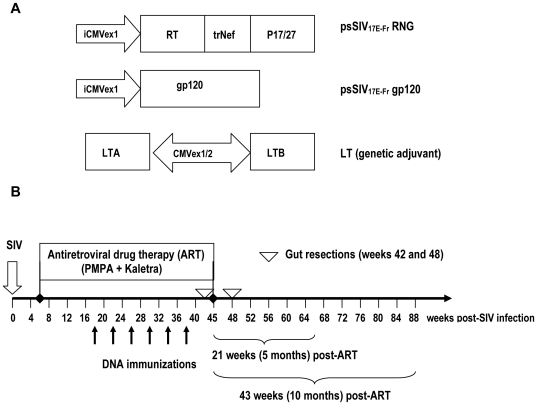
Study Design and DNA vaccines. (**A**) *DNA vaccines*. The unadjuvanted DNA vaccine consisted of a co-delivery of 2 plasmids, one encoding a fusion of SIV RT/Nef/Gag (RNG) and a second encoding SIV gp120 (SIV_17E-Fr_). The DNA+LT vaccine is a co-delivery of the RNG and SIVgp120 plasmid and a 3^rd^ plasmid encoding the A and B subunits from the heat-labile enterotoxin of E. Coli (LT) downstream from 2 separate CMV promoters. The DNA group received 32 µg of the DNA vaccine (16 µg per plasmid) per dose. The DNA+LT group received 32 µg of the DNA vaccine plus 3.2 µg of the LT genetic adjuvant. (**B**) *Study Design*. Anti-retroviral drug therapy (ART) consisting of lopinavir/ritonavir (Kaletra) and PMPA was started 6 weeks after intravenous infection with SIV/DeltaB670 and continued until week 45 post-infection. Arrows indicate time-points of 6 DNA immunizations, administered one month apart. Inverted open triangles indicate time-points when a gut jejunal resection was performed and lymphocytes and tissues isolated for analysis of viral replication, immune responses, and CD4+ T cell counts in the gut. ART was discontinued 7 weeks after the last DNA immunization to assess the effects of vaccination on protecting against viral rebound and disease. Vaccine efficacy, defined by containment of viral rebound was assessed at 5 (66 weeks post-infection) and 10 (88 weeks post-infection) months after stopping ART.

An effective therapeutic HIV vaccine will likely need to induce CD4+ and CD8+ T cell responses against multiple antigens to be effective in humans infected with diverse HIV subtypes. We hypothesized that a vaccine capable of enhancing these responses in the gut associated lymphoid tissues (GALT) would be critical for effective immunotherapy because it would target the primary site of virus production. Toward this end, we constructed a PMED SIV DNA vaccine consisting of a plasmid expressing codon-optimized RT, Nef, and Gag from SIV (17E-Fr) [23] as a single fusion protein co-delivered with a second plasmid expressing SIVgp120 (Fig. 1A). To enhance mucosal responses, a third plasmid was included that expressed a mucosal adjuvant consisting of the A and B subunits of *E.coli* enterotoxin (LT) [24], [25], [26] (Fig. 1A). We previously showed this adjuvant substantially increased CD4+ and CD8+ T cell responses when compared to responses induced by unadjuvanted PMED DNA vaccines [26].

Macaques were infected intravenously with SIV/DeltaB670, a highly pathogenic primary isolate that induces AIDS in most rhesus macaques within 5–17 months post-infection with a mean time to AIDS of 11 months [27]. Because the consensus sequence of *env* in the SIV/DeltaB670 inoculum is 15% divergent from the *env* sequence in the vaccine [23], this study mimics therapeutic immunization of humans infected with diverse variants within a given HIV subtype.

ART (PMPA+lopinavir/ritonavir) was initiated six weeks post-infection (Fig. 1B). Macaques infected with SIV/DeltaB670 exhibit more rapid immune dysfunction, disease progression, and shorter survival time when compared to humans infected with HIV [28], [29], [30], [31], As such, initiating ART 6 weeks post-infection with SIV is analogous to initiating HAART in HIV-infected humans at later time points during the chronic stage of infection. In both situations, the viral setpoint has occurred and disease progression, defined by progressive declines in CD4 lymphocytes, is evident. In this study, by 6 weeks post-infection, the viral setpoint was reached and animals were already exhibiting a mean 33% loss in CD4 counts relative to baseline levels.

All animals received ART alone for 12 weeks (weeks 6–18) to allow the drugs to establish maximum suppression of viremia prior to vaccination (Fig. 1B). During weeks 18–38 of the study, each group continued to receive uninterrupted ART plus 6 monthly doses of either a PMED SIV DNA vaccine (SIV *RT-nef-gag*+*env*) alone (DNA group, N = 14) or co-delivered with LT (DNA+LT group, N = 14) or empty plasmid (CONTROL group, N = 13). ART was then withdrawn 6 weeks after the final immunization (week 45 post-infection), and the effect of each vaccine on virus-specific immunity, virus burden, prevention of viral rebound, and protection from AIDS for 5 and 10 months after stopping ART was determined (Fig. 1B).

### Antiretroviral drug therapy with PMPA and Kaletra affords a highly variable impact on virological control in SIV-infected macaques

**Figure 2:**
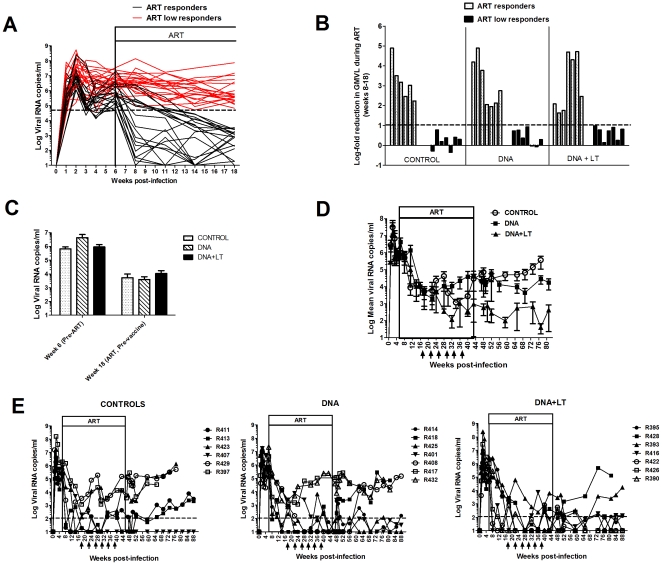
Therapeutic DNA vaccination reduces viral load and protects from viral rebound after drug is stopped. Plasma viral RNA load was determined by real time RT-PCR. Boxed areas (ART) indicate period of antiretroviral drug therapy. Arrows indicate DNA vaccine doses. (**A**) Response to ART (weeks 0–18). Shown are viral loads in 41 SIV-infected macaques before starting ART (weeks 0–6) and during the first 12 weeks of ART only (weeks 6–18). ART responders (black) had declining viral loads during ART only. ART low responders (red) had little or no decline in viral loads during ART only and maintained ≥5×10^4^ viral RNA copies/ml (dotted line). (**B**) Log-fold reduction in viral load during ART only. The change in viral load during ART in each animal was determined by dividing the mean viral load prior to initiating ART (weeks 4–6) by the mean viral load during the first 12 weeks of ART but prior to initiating the therapeutic vaccinations (weeks 8–18). Shown is the log-fold reduction in mean viral load during ART for each animal. ART low responders had <1 log-fold reduction. ART responders exhibited 1.6–4.9 log-fold reductions in viral load during ART. (**C**) Mean plasma viral loads in each group prior to initiating ART (week 6, Pre-ART) and after 12 weeks on ART but before starting therapeutic vaccinations (week 18, Pre-vaccine). (**D**) Mean plasma viral loads during vaccinations and after withdrawing ART. Differences in mean viral loads after withdrawing ART are significant starting at weeks 68 and 48 in the DNA (P = 0.019) and DNA+LT (P = 0.002) groups, respectively, and remained significant thereafter when compared to controls (Wilcoxon test). (**E**) Viral loads in each animal. Protection from viral rebound was defined as containment of viral load ≤100 viral RNA copies/ml (hashed line) for a period of at least 5 months after drug was withdrawn (weeks 45–66).

Similar to previous studies [7], [32], the virological response to ART even before the vaccinations were started was highly variable with only 20 of the 41 animals showing suppression of plasma viral load within 12 weeks after starting ART (ART responders, Fig. 2A). The remaining 21 macaques showed little or no response to ART (ART low responders, Fig. 2A). These animals maintained persistently high viral loads (>5×10^4^) and exhibited <1 log-fold reduction in mean viral load during the first 12 weeks of ART treatment alone (weeks 7–18) when compared to the mean viral loads measured prior to initiation of ART (weeks 1–6) (Fig. 2B). The majority of these animals (16/21) were sacrificed prior to week 66 due to progression to AIDS. In contrast, animals responding well to ART suppressed viral loads below 10^4^ viral RNA copies/ml within 12 weeks after starting drugs (Fig. 2A) and exhibited a 1.6–4.9 log-fold reduction in mean viral load during ART when compared to pre-ART levels (Fig. 2B). Host and virological factors that may have influenced the response to ART in this study are currently under investigation and will be reported elsewhere. However, we and others have already shown that therapeutic vaccination is not effective in animals failing ART [7], [32]. Because the goal of this study was to determine the impact of therapeutic vaccination in combination with an effective ART regimen, animals failing ART were therefore excluded from this evaluation. After excluding ART failures, there were 6 animals remaining in the control group, 7 in the DNA group, and 7 in the DNA+LT group (Fig. 2B).

### DNA vaccines lower viral burden and prevent viral rebound post-ART

Among ART responders, there were no significant differences in mean viral loads between the groups before starting ART (week 6) or before initiating the therapeutic vaccinations during ART (week 18) (Fig. 2C). During the DNA vaccinations and prior to withdrawing ART, the mean viral load in the DNA+LT group declined to <10^3^ copies/ml after the 3^rd^ vaccine dose and remained below this level by the end of the vaccine/ART treatment period (week 45) (Fig. 2D). After stopping ART at week 45, mean viral load in the control group increased and reached >10^5^ copies/ml by the end of the study whereas the mean viral load in the unadjuvanted DNA vaccine group did not increase resulting in a 5–15-fold lower viral burden post-ART when compared to the control group (P = 0.0078) (Fig. 2D). In striking contrast, the DNA+LT vaccine group continued to maintain mean viral loads below 10^3^ copies/ml for over 8 months (weeks 47–81) after stopping ART (Fig. 2C), a level that represents a substantial 2–4 log-fold reduction in mean viral load post-ART when compared to controls (P = 0.0023)

Containment of viral loads at levels at or below 100 viral RNA copies/ml for a period of at least 5 months post-ART (weeks 45–66) was selected as the criterion for therapeutic efficacy in protection from viral rebound because SIV-infected rhesus macaques that are elite controllers maintain viremia below this level [33] and because we previously showed that even transient (i.e. 3–6 months) containment of viremia below this level after stopping ART translated to longer-term (>1 year) protection from disease progression in macaques infected with this virus [32]. Prior to initiating therapeutic immunizations at week 18, only 2/6 animals in the control group, 1/7 in the unadjuvanted DNA vaccine group, and 2/7 in the DNA+LT group had virus loads ≤100 copies/ml. After another 37 weeks on ART and the addition of 6 therapeutic DNA immunizations (weeks 18–45), the number of animals in the control group with virus loads ≤100 copies/ml remained unchanged (2/6) whereas in the DNA and DNA+LT vaccine groups, the number had increased to 5/7. After ART was stopped, 1/6 (16.7%) animals in the control group, 3 of 7 (42.9%) in the DNA group, and 6 of 7 macaques (86%) in the DNA+LT group controlled virus load below 100 copies/ml for a period of at least 5 months post-ART (week 45–66). Furthermore, viremia was maintained at these very low levels (≤100 copies/ml) without further intervention in all but one animal (R393, DNA+LT group) for 10 months after stopping ART (until week 88, the duration of the study). In 5 macaques; including 1 control, 1 in the DNA group, and 3 in the DNA+LT group; plasma viremia declined to undetectable levels (<3 copies/ml) by the end of the study.

**Figure 3:**
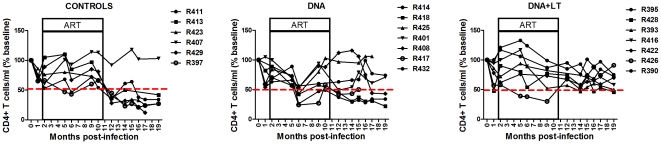
Therapeutic DNA vaccination protects from AIDS. Maintenance of CD4+ T cell counts as a marker of clinical disease progression was measured by flow cytometry. Shown is % CD4+ T cell count relative to baseline. Animals that maintained their CD4+ T cell counts above 50% of their baseline level (hashed line) level remained clinically healthy throughout the study.

CD4 counts in the blood paralleled virus burden in plasma in that most animals showing viral rebound within the first 5 months after stopping ART exhibited CD4 decline by the end of the study to levels that were below 50% of the baseline level measured prior to infection whereas all animals that contained viral rebound for at least 5 months after drug was stopped showed stable CD4+ lymphocyte counts for the duration of the study (Fig. 3). Interestingly, the DNA+LT vaccine protected all 7 animals from CD4 decline for the full duration of the study after stopping ART including 2 animals that either exhibited early (R428) or delayed (R393) viral rebound after stopping ART (Fig. 2E). This suggests that transient containment of viral rebound or other vaccine-induced effects may afford a therapeutic benefit. Together, these results show that DNA vaccination during effective ART protected rhesus macaques chronically infected with SIV from viral rebound and this effect translated into durable protection from progression to AIDS. Importantly, the LT adjuvant appears to be a critical component of this effect since only the DNA+LT vaccine afforded significant protective efficacy when compared to controls from viral rebound and/or disease progression after drug was withdrawn (Table 1).

**Table 1:** Protection from viral rebound and CD4 decline.

Group	Protection from viral rebound^1^		Protection from CD4 decline^2^	
	# protected	*P* 3	# protected	*P* 3
**CONTROLS**	1/6		1/6	
**DNA vs. CONTROLS**	3/7	0.559	3/7	0.559
**DNA+LT vs. CONTROLS**	6/7	0.025	7/7	0.005

1 Containment of plasma viral load ≤100 RNA copies/ml for a period of at least 5 months (weeks 45–66) after stopping ART

2 Maintenance of CD4 counts at or above 50% baseline levels for the duration of the study after stopping ART

3 P values were determined by Fisher's exact test. After adjusting for 2 comparisons to the control group, a P value of 0.05/2 = 0.025 is considered significant.

### Relationship between TRIM5 or MHC class I haplotypes and therapeutic outcome

Previous studies have shown that polymorphisms in the viral resistance factor, *TRIM5*, can influence susceptibility to SIV [34], [35]. To determine if this host resistance factor influenced our results, the *TRIM5* alleles that have been associated with intermediate susceptibility (TFP/Q and Q/CypA) or resistance to SIVmac251 or SIVsmE660 infection (TFP/TFP and TFP/CypA) were determined. Table 2 shows that 15 of the 20 animals in this study had either TFP/TFP or TFP/CypA (resistant) genotypes and the remaining 5 had TFP/Q or Q/CypA (intermediate susceptibility). Analysis of *TRIM5* genotype versus therapeutic outcome shows that the number of animals with the resistant *TRIM5* genotypes that exhibited elite control of virus was not significantly different from the number that failed to control viral rebound (Table 3). This result indicates *TRIM5* factors reported to be associated with enhanced resistance to SIVmac251 or SIVsmE660 do not have a similar influence on the control of SIV/DeltaB670.

**Table 2:** Relationship between *TRIM5* haplotype and outcome.

Group	Animal	Outcome	*TRIM5*
**CONTROLS**	R411	Rebound^1^	TFP/Q^4^
	R413	Rebound	TFP/TFP^5^
	R423	Rebound	TFP/TFP
	R407	Elite controller^2^	TFP/TFP
	R429	Rebound	Q/CypA^4^
	R397	Rebound	TFP/Q
**DNA**	R414	Elite controller	TFP/TFP
	R417	Rebound	TFP/TFP
	R418	Rebound	TFP/Q
	R425	Elite controller	TFP/Q
	R401	Elite controller	TFP/TFP
	R408	Rebound	TFP/TFP
	R432	Rebound	TFP/TFP
**DNA+LT**	R393	Late rebound^3^	TFP/CypA^5^
	R395	Elite controller	TFP/TFP
	R422	Elite controller	TFP/TFP
	R428	Rebound	TFP/TFP
	R416	Elite controller	TFP/TFP
	R426	Elite controller	TFP/TFP
	R390	Elite controller	TFP/TFP

1 Rebound: Plasma virus load rebounded to >1000 copies/ml within 26 weeks after stopping ART.

2 Elite Controller: plasma virus load is contained post-ART at ≤100 copies/ml for duration of study (44 after stopping ART).

3 Late Rebound: Plasma virus load was contained for at least 26 weeks after stopping ART but rebounded to >1000 copies/ml before end of study.

4 TFP/Q and Q/CypA: associated with intermediate susceptibility to SIVmac251 and SIVsmE660 infection (34, 35).

5 TFP/TFP and TFP/CypA: associated with resistance to SIVmac251 and SIVsmE660 infection (34, 35).

**Table 3:** Statistical Analysis of Trim5 haplotype versus outcome.

Phenotype	Intermediate susceptibility (TFP/Q or Q/CypA)		Resistance (TFP/TFP or TFP/CypA)	
Outcome	Rebound	Elite controller	Rebound	Elite controller
**Number of animals**	4/5	1/5	6/15	9/15
**P value** 1	0.206		0.466	

1 2-sided Fisher's Exact Test.

Macaques were also typed for currently known MHC class I alleles including Mamu-A*01 and Mamu-B*17 that have been associated with slower disease progression in rhesus macaques infected with SIVmac239 or mac251 [36]. Among the 4 animals positive for the A*01 or B*17 alleles, 3 animals (2 positive for A*01 in the DNA group and 1 positive for B*17 in the control group) failed to control viral rebound after ART was withdrawn indicating A*01 and B*17 alleles did not provide an advantage against SIV/DeltaB670 (not shown). This result is consistent with our previous study where we showed no significant difference in viral control between A*01 positive and negative rhesus macaques infected with SIV/DeltaB670 [32].

### DNA vaccines increase virus-specific CD4+ and CD8+ T cell responses, but not antibody responses, in the blood

**Figure 4:**
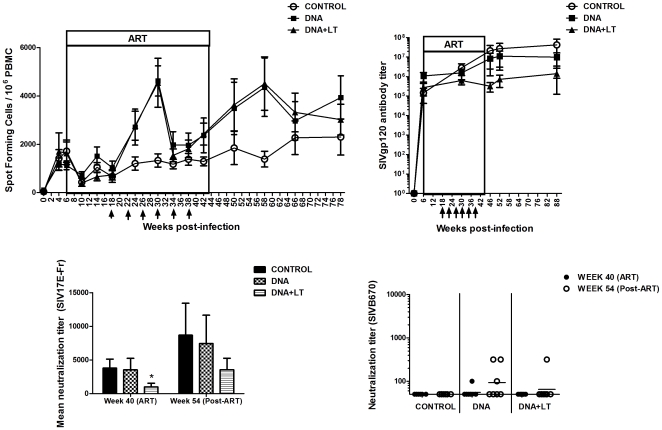
Therapeutic DNA vaccines increase SIV-specific IFN-γ T cell responses, but not antibody, in the blood. (**A**) IFN-γ T cell responses were measured throughout the study by ELISPOT. Shown is the mean (± SEM) SIV-specific IFN-γ T cell response at each time-point minus background. Background levels were <25 SFC/10^6^ PBMC. Differences between the DNA or DNA+LT vaccine groups and controls were significant (P≤0.0063, repeated measures ANOVA). (**B**) SIV-gp120 specific binding antibody was measured throughout the study by ELISA. Shown are mean (± SEM) endpoint titers. Mean antibody titers in the DNA+LT group were significantly lower (P = 0.0422) than the controls (**C**) Neutralizing antibody responses were measured against the vaccine strain (SIV/17E-Fr). Shown are mean (± SEM) neutralization titers after the final DNA vaccine dose but before stopping ART (week 40) and 9 weeks after stopping ART (week 54). *P = 0.0292 (**D**) Neutralizing antibody titers in each animal before (week 40) and after (week 54) stopping ART against the challenge strain (SIV/DeltaB670).

To determine the impact of the vaccines on the overall magnitude of the combined CD4/CD8+ T cell response, SIV-specific IFN-γ responses were measured by ELISPOT throughout the study. IFN-γ T cell responses prior to DNA vaccination (week 18) were comparable between all 3 groups but increased to significantly higher levels in both vaccine groups following adminstration of the 2^nd^ (week 24) and 3^rd^ (week 30) DNA vaccine doses. IFN-γ T cell responses then declined but subsequently increased in all 3 groups after stopping ART (Fig. 4A). Significantly, both vaccine groups developed higher IFN-γ T cell responses after stopping ART than the controls even though mean virus loads post-ART in the control group were higher. These results suggest the therapeutic vaccinations primed the animals to develop a stronger recall T cell response after ART was stopped.

To determine if therapeutic vaccination influenced antibody responses, SIVgp120-specific binding antibody was measured at several time-points throughout the study by ELISA. In contrast to the T cell response (Fig. 4A) mean antibody responses in both vaccine groups were lower than in the controls (Fig. 4B). Neutralizing antibody responses against the vaccine strain (17E-Fr) measured before (week 40) and 10 weeks after (week 54) ART was stopped were consistent with the ELISA results in that the DNA+LT group, which had the lowest mean viral burden, also had the lowest mean neutralizing antibody response (Fig. 4C). As expected, higher mean ELISA antibody titers in each animal positively correlated with higher mean viral loads between weeks 30–88 (P = 0.0206, Spearman's correlation test), a result that indicates the level of viral replication and not vaccination, had a greater influence on the antibody response. Only 3 vaccinated animals had very low cross-neutralizing antibody against the challenge strain (B670) either before (week 40) or after stopping ART (week 54) (Fig. 4D), a result that further indicates serum antibody likely had little impact on viral control. Despite this evidence, mucosal secretions were not collected in this study so a possible role for mucosal antibody in viral control cannot be entirely excluded.

**Figure 5:**
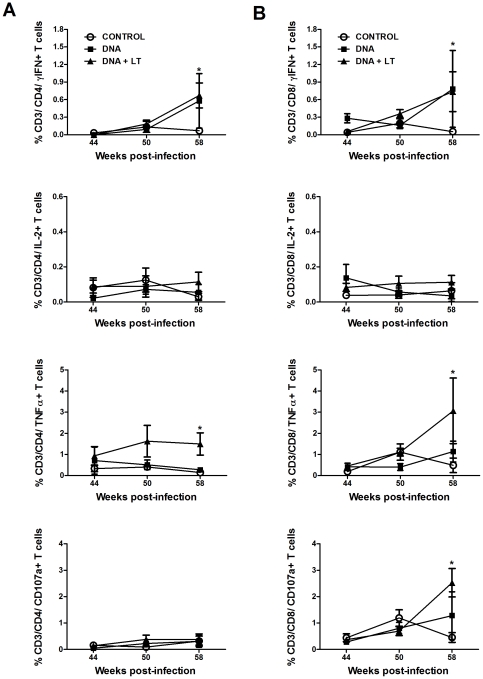
Therapeutic DNA vaccination increases CD4+ and CD8+ T cell responses in the blood after stopping ART. SIV-specific CD4+ and CD8+ T cells with cytokine (IFN-γ, TNF-α, IL-2) or cytolytic (CD107a) effector functions were measured in PBMC at 3 time-points just before (week 44) and after (weeks 50, 58) ART was withdrawn by flow cytometry following *in vitro* stimulation with overlapping peptide pools (RT, Nef, Gag, and Env) derived from the SIV genes included in the DNA vaccine. Shown is the mean frequency and functional nature of the SIV-specific effector T cell response in the CD4+ (**A**) and CD8+ (**B**) T cell subsets. *indicates P<0.05 in only the DNA+LT group.

SIV-specific CD4+ and CD8+ T cell responses were further characterized in the blood by flow cytometry to determine the impact of the therapeutic DNA vaccines on separate CD4+ and CD8+ T cell subsets and on multiple T cell effector functions, including secretion of IFN-γ, TNF-α, and IL-2 cytokines and expression of CD107a, a marker for cytolytic function [37]. We focused analysis on responses just before as well as the first 14 weeks after ART was withdrawn (weeks 44–58) to determine if there was a relationship between T cell responses present shortly before or after stopping ART and the initial control of viral rebound. Similar to the IFN-γ ELISPOT results, both vaccine groups developed elevated SIV-specific IFN-γ CD4+ (Fig. 5A) and CD8+ (Fig. 5B) T cell responses by 14 weeks after ART interruption (week 58). However, after stopping ART, only the DNA+LT group developed higher frequencies of SIV-specific CD4+ and CD8+ T cells secreting TNF-α (Fig. 5A and 5B) and CD8+ T cells expressing the cytolytic marker CD107a (Fig. 5B).

**Figure 6:**
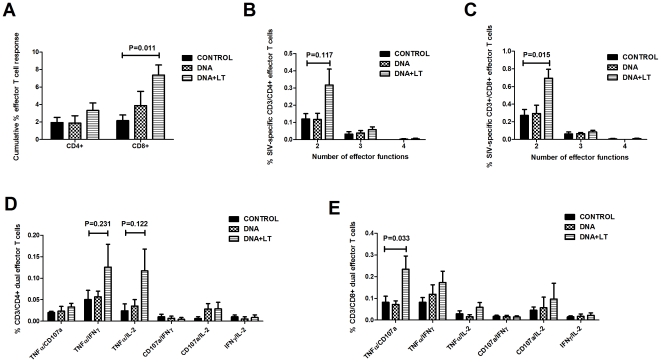
LT-adjuvanted DNA vaccine increases T cells with multiple effector functions in the blood. SIV-specific T cells with 1–4 cytokine (IFN-γ, TNF-α, IL-2) or cytolytic (CD107a) effector functions were measured in PBMC 14 weeks after ART was withdrawn (week 58) by flow cytometry following *in vitro* stimulation with overlapping peptide pools derived from genes included in the DNA vaccine (RT, Nef, Gag, Env). Boolean gating was performed to identify the total frequency of CD4+ and CD8+ T cells in the blood of ART responders with any one of 1–4 effector functions. (**A**) Cumulative mean (±SEM) frequency of SIV-specific CD4+ and CD8+ T cells expressing IFN-γ, TNF-α, IL-2 or CD107a. Cumulative mean frequency (± SEM) of SIV-specific (**B**) CD4+ and (**C**) CD8+ T cells co-expressing any combination of 2–4 effector functions. Mean (± SEM) frequency of SIV-specific (**D**) CD4+ and (**E**) CD8+ T cells expressing the indicated combination of dual effector functions.

The overall magnitude of the T cell effector response at week 58 was further analyzed by measuring the frequency of SIV-specific T cells expressing any one of the three cytokines (IFN-γ, TNFα, IL-2) or CD107a. The DNA+LT vaccine had minimal impact on the CD4+ T cell response but significantly increased SIV-specific CD8+ T cell responses when compared to controls (*P* = 0.011) (Fig. 6A). Boolean analysis of co-expression of combined effector functions showed very few CD4+ (Fig. 6B) or CD8+ T cells (Fig. 6C) in the blood with more than 2 effector functions. However, the DNA+LT vaccine significantly increased CD8+ T cell responses (P = 0.015) with dual effector functions (Fig. 6C). The DNA+LT vaccine increased SIV-specific CD4+ T cells with dual TNF-α/IFN-γ and TNF-α/IL-2 functions but these trends fell short of statistical significance (*P*>0.122, Fig. 6D) and significantly increased the frequency of CD8+ T cells with dual TNF-α and cytolytic effector functions (P = 0.033) (Fig. 6E). These data suggest that viral control after stopping ART in the DNA+LT group may be due, in part, to enhanced induction of cytotoxic T lymphocytes with multiple effector functions that are capable of targeting and eliminating infected cells.

### DNA vaccines reduce virus production in lymph nodes and gut

**Figure 7:**
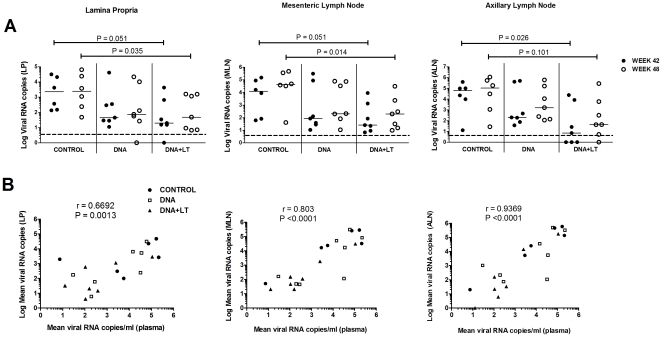
Therapeutic DNA vaccination reduces virus production in the lymph nodes and gut mucosa. (**A**) Viral load in gut lamina propria (LP), mesenteric lymph nodes (MLN), and axillary lymph nodes (ALN) in each animal 3 weeks before (week 42, solid circles) and 3 weeks after ART was withdrawn (week 48, open circles). Solid lines indicate the median viral load for the group. Hashed line indicates the lower limit of detection for this analysis (3 viral RNA copies). (**B**) Correlations between mean virus production in the gut lamina propria (left panel), mesenteric lymph nodes (middle panel) and axillary lymph nodes (right panel) vs. mean plasma viral load in each animal post-ART. Each symbol corresponds to mean virus load in the plasma of each animal after stopping ART (weeks 47–88) on the X axis vs. mean virus production measured in tissues at weeks 42 and 48 on the Y axis. Spearman's rank correlation coefficients are shown.

During ART, lymphoid tissues, particularly those in the GALT, serve as primary sites for virus production [1]. To determine the impact of immunization on tissue-associated virus production, full-length transcripts prepared for packaging into virions were quantified in mononuclear cells purified from axillary (ALN) and mesenteric lymph nodes (MLN) and the gut lamina propria (LP) 3 weeks before (week 42) and 3 weeks after (week 48) discontinuing ART. At both time points, the unadjuvanted DNA vaccine and DNA+LT vaccine groups showed a 2–3 log-fold reduction in median virus production in all 3 tissues when compared to the controls but differences were significant only in the DNA+LT group (P≤0.051) (Fig. 7A). Since viral rebound did not occur in controls or vaccine failures until after week 52 (Fig. 2E), viral loads measured in the tissues post-ART at week 48 in each animal were similar to levels measured prior to stopping ART at week 42 (Fig. 7A). For each animal, the mean viral load in tissues at weeks 42 and 48 strongly correlated (*P*<0.0013) with the mean plasma viral load measured after drug was stopped (weeks 48–88) (Fig. 7B). Importantly, the consistently lower levels of virus production in all 3 tissues of macaques immunized with the DNA+LT vaccine strikingly corresponded to this vaccine being more effective in substantially reducing mean viral load and preventing viral rebound after stopping ART (Fig. 2D and 2E). These results indicate that the DNA+LT vaccine elicited responses that targeted and reduced virus persisting in the gut and lymph nodes during ART and suggest that reducing virus production in the tissues may be important for protection from viral rebound after cessation of ART.

### Viral control correlates with a broader CD8+ T cell response detectable in the GALT but not the blood

**Figure 8:**
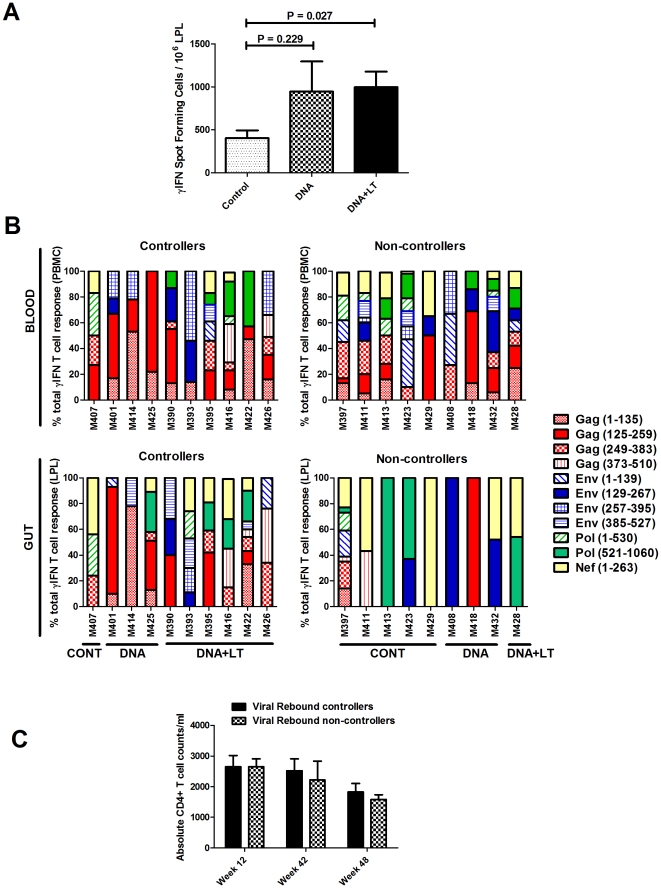
Therapeutic DNA vaccination increases mucosal SIV-specific T cell responses in the gut. (**A**) The magnitude of the SIV-specific IFN-γ T cell response in the gut was determined by ELISPOT analysis of lamina propria lymphocytes (LPL) isolated from jejunal resections. Results represent the mean number of IFN-γ spot forming cells (± SEM) for each group from 2 separate experiments performed at 2 time-points post-infection (weeks 42 and 48). (**B**) Breadth of the T cell response in each macaque in the blood (top panels, PBMC) and gut (lower panels, GALT). SIV-specific T cell responses in PBMC and LPL isolated from the jejunum were measured by IFN-γ ELISPOT assay against 11 separate pools of overlapping peptides (15-mers overlapping by 11 amino acids) comprising the indicated amino acid sequences from SIV Gag, Env, Pol, and Nef. The percent contribution of each peptide-pool specific response to the total response was determined by dividing the mean number of IFN-γ spot forming cells (SFC) measured against each individual peptide pool by the sum of the response against all peptide pools. Results represent the average of 2 time-points tested at weeks 42 and 48. (**C**) Mean absolute GALT CD4+ T cell counts measured by flow cytometric analysis of mononuclear cells isolated from the jejunal lamina propria at weeks 12, 42, and 48 post-infection were similar in animals that controlled vs. failed to control viral rebound after ART was stopped. Controllers are animals that contained virus at ≤100 copies for at least 5 months after stopping ART. Non-controllers are animals that exhibited viral rebound within the same timeframe.

To determine the impact of therapeutic DNA vaccination on mucosal responses in the GALT, SIV-specific T cell responses in the lamina propria of the small intestine were measured. Because there was no difference in viral loads at weeks 42 and 48 (Fig. 7A), mucosal responses measured in each animal at these two time-points were averaged. The magnitude of the mucosal T cell response in ART responders was determined by measuring the frequency of SIV-specific T cells expressing any one of the three cytokines, IFN-γ, TNF-α, IL-2, or the cytolytic marker, CD107a. Boolean analysis of these effector functions in combination revealed no significant differences among the three groups in the frequency of SIV-specific CD4+ or CD8+ effector T cells in the gut, and very few CD4+ or CD8+ T cells in this compartment demonstrated more than 2 effector functions (not shown). However, both vaccines increased IFN-γ CD4+ and CD8+ T cell responses measured by either ELISPOT (Fig. 8A) or intracellular cytokine staining (ICS, not shown) in the gut when compared to the controls, but only in the DNA+LT group was this response statistically significant (*P* = 0.027). These data indicate that vaccination increased IFN-γ T cell responses in the gut but had no significant impact on the frequency of mucosal CD4+ or CD8+ T cells with other effector functions.

The breadth of the T cell response in the blood and gut mucosa against viral antigens included in the vaccine was also measured by IFN-γ ELISpot. T cell responses were mapped against separate pools of overlapping peptides spanning the entire sequence encoded by the vaccines and the responses detected against each peptide pool at weeks 42 and 48 were averaged. No clear difference in the breadth of the T cell response was observed in the blood of animals that controlled viral rebound versus noncontrollers (Fig. 8B, top panels). The T cell repertoire in the GALT, however, was strikingly distinct from that observed in the blood. Animals that failed to control rebound exhibited a limited T cell repertoire in the GALT against only 1–3 peptide pools; and responses were dominanted by recognition of Nef (yellow). In contrast, animals that controlled viral rebound developed a broader response in the gut against 4–9 peptide pools that were dominated by responses against Gag (red) (Fig. 8B, lower panels). This difference was not due to greater preservation of CD4+ T cells in the gut of the animals that controlled viral rebound because CD4 counts in the gut at these time-points were similar between viral rebound controllers and non-controllers (Fig. 8C).

**Figure 9:**
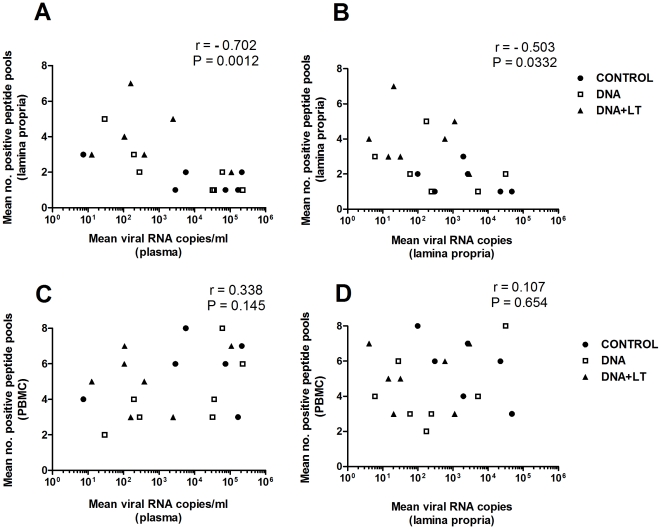
Viral control correlates to CD8+ T cell responses with broad specificity in the gut. Correlations between the breadth of the T cell response in the gut (**top panels A and B**) or the blood (**lower panels C and D**) vs. viral loads in blood (**left panels A and C**) and gut (**right panels B and D**). Each symbol corresponds to mean virus load in the plasma of each animal after stopping ART (weeks 47–88) or gut (weeks 42, 48) on the X axis vs. mean number of peptide pools recognized in the IFN-γ ELISPOT assay at weeks 42 and 48 in the blood or gut lamina propria on the Y axis. Spearman's rank correlation coefficients are shown.

These findings demonstrate that a highly diverse T cell response localized in the GALT may play an essential role in the prevention of viral rebound and disease progression post-ART by affording immune control of virus production within the primary reservoir of infected cells. To investigate this further, we examined the correlation between the breadth of the T cell response present in the blood or gut and mean viral loads in plasma after ART was stopped (weeks 45–88) or in tissues (weeks 42, 48). Figure 9 shows a significant correlation between a higher number of peptide pools recognized by T cells in the gut and lower viral replication in the plasma post-ART (r = −0.703, P = 0.0012, Fig. 9A) or gut lamina propria (r = −0.503, P = 0.0332, Fig. 9B). Similar results were seen when the breadth of the T cell response in the gut was compared to viral replication in other compartments including PBMC (r = −0.525, P = 0.0251), axillary lymph node (r = −0.490, P = 0.0392), and mesenteric lymph node (r = −0.523, P = 0.0258) (not shown). In contrast, the breadth of the T cell response in the blood did not correlate with lower viral loads in blood (Fig. 9C), the lamina propria (Fig. 9D) or other compartments including PBMC, axillary and mesenteric lymph nodes (not shown). The overall magnitude of the IFN-γ T cell response determined by ELISPOT did not correlate with lower viral loads in plasma or tissues (r≥−0.117; P≥0.622) (not shown) suggesting that increasing the breadth of the mucosal T cell response against multiple viral antigens in the gut had more effect on controlling viral replication than increasing the overall magnitude of the mucosal T cell response. Taken together, the strong correlation between protection from viral rebound and a broad specificity in the T cell repertoire detected in the gut, but not the blood, emphasizes that immune responses in the gut mucosa may provide a better correlate of vaccine-induced immunity and therapeutic efficacy.

## Discussion

Highly active antiretroviral therapy (HAART) can reduce and maintain viral load at <10 HIV-1 RNA copies/ml of plasma indefinitely. However, HAART does not result in a cure and if drug treatment is interrupted, virus rebound usually occurs within weeks. To achieve long-term control of HIV-1 in the absence of ART (functional cure) or complete eradication of the persistent virus (cure), new therapies are therefore needed that can be used as an adjunct to HAART to target the latent HIV-1 and occult viral reservoirs.

Toward this end, we previously showed that an epitope-based PMED DNA vaccine administered during PMPA monotherapy significantly increased T cell responses in the blood of SIV-infected macaques and induced durable containment of viral rebound in those animals that responded well to PMPA [32]. However, the positive outcome in this study was likely due, in part, to initiating drug within two weeks after infection when the immune system was less compromised.

In the present study, we sought to determine if PMED DNA immunization would be as effective when drug was initiated later during chronic infection. In these studies, we also investigated the impact of co-administering a plasmid expressing the potent mucosal adjuvant LT (A and B subunits of the heat-labile enterotoxin from *E. coli*) with the PMED DNA vaccine to investigate the hypothesis that increasing virus-specific responses at the site of the majority of virus production, the gut, would improve therapeutic efficacy. Consistent with our previous study, we found that both the unadjuvanted and LT-adjuvanted DNA vaccines augmented the magnitude of the T cell responses and reduced viral load in animals that responded well to ART. However, immunization with the LT-adjuvanted vaccine afforded an unprecedented 3–5 log-fold reduction in plasma viral load and superior long-term protection from viral rebound and progression to AIDS after ART was stopped.

This result is in contrast to previous therapeutic vaccine approaches tested in macaques as an adjunct to ART during chronic SIV infection including DNA vaccines administered by other delivery methods [9], [38], [39], peptide-pulsed cells [5], [6] and viral vectored vaccines [40]. These approaches afforded only transient control of initial viral rebound, less than 2 log-fold reductions in mean viral load post-ART [5], [6], [9], [38], [40] or, despite increasing immune responses, had no impact on virus burden [39]. Although therapeutic outcomes are influenced by a number of variables such as viral strain, timing of intervention, and effectiveness of the antiretroviral drugs, our data suggest that the more profound suppression of virus and durable protection from viral rebound and disease with the LT-adjuvanted PMED DNA vaccine may be due to the ability of PMED to induce mucosal responses [22], [41], [42] and the effects of LT in augmenting these responses. Indeed, we found that containment of viral rebound correlated with a significant reduction in viral replication in the gut and lymph node reservoirs and the induction of a diverse anti-viral CD8+ T cell response in the GALT but not the blood, a finding that provides the first evidence that vaccine induction of mucosal responses during therapy can target and reduce persistent virus in the gut.

Both the unadjuvanted and LT-adjuvanted DNA vaccines similarly increased SIV-specific IFN-γ T cell responses in the blood and gut, but the magnitude of the IFN-γ T cell response did not predict protection from viral rebound. Analysis of other possible immune effectors of viral control revealed, however, that improved viral control in the LT-adjuvanted vaccine group significantly correlated with a broader specificity in the IFN-γ T cell repertoire in the GALT that included increased T cell recognition of Gag. These results indicate that specificity, rather than the magnitude, of the IFN-γ T cell response may be a more critical factor for controlling virus production in the mucosa.

Improved viral control in the LT-adjuvanted DNA vaccine group was also associated with a higher frequency of CD8+ T cells with dual TNF-α and cytolytic (CD107a) effector functions in the blood. Direct cytotoxicity was not measured in this study but expression of CD107a is a marker of degranulation that has been validated as a reliable surrogate for CD8+ T cells with cytolytic effector function [37]. The induction of cytolytic effectors is critical to control of virus because these cells can target and eliminate persistently infected cells. Consistent with this possibility, prophylactic vaccine studies in rhesus macaques have shown strong correlations between control of SHIV or SIV infections and CD8+ T cells with a strong cytolytic phenotype but less IFN-γ expression in the blood [43] and mucosa [27], [44].

Interestingly, the association between improved viral control in the DNA+LT group and a higher frequency of CD8+ T cell responses with dual TNFα/CD107a effector functions was seen in the blood, but not the gut. In contrast, the broad IFN-γ T cell response in the gut mucosa that correlated with protection from viral rebound appeared to be sequestered in the gut because the repertoire differed in specificity from IFN-γ T cell responses measured in the blood at the same time-points. These results show that immune responses measured in the periphery do not mirror immune correlates of viral control in the mucosa.

The mechanisms underlying the differential compartmentalization of T cell specificity and function in the blood versus gut are not known. Following skin immunization, antigen-presenting cells expressing skin-derived antigen can migrate to the mucosa and induce T cell responses that remain localized in the gut [45], [46]. In this manner, presentation of skin-derived antigen in the gut would induce a broader antigen-specific T cell repertoire that would be distinct from the repertoire in the blood. Furthermore, DNA vaccines alone or in combination with viral vaccines are known to increase the breadth [8], [32], [47] and functional avidity of the T cell response [48]. These effects, when combined with the fact that vaccination to the skin induces mucosal immune responses [24], [25], [41], [45], [49], [50], [51], may act in concert to induce a broad repertoire of high-avidity CTLs localized in the gut. These effectors would be more effective in clearing residual virus-infected cells since high avidity CTLs are known to be more effective than low avidity CTLs in clearing viral infections [27], [46], [52], [53].

Taken together, the results indicate multiple mechanisms likely contributed to the enhanced efficacy observed with the LT-adjuvanted DNA vaccine. The higher frequency of SIV-specific CD8+ T cell responses with TNF-α and cytolytic effector functions induced by the LT-adjuvanted DNA vaccine may have contributed to improved viral control by enhancing clearance of infected cells. The broader repertoire of SIV-specific T cell responses in the gut of animals immunized with the LT-adjuvanted vaccine may have further improved protection from viral rebound by creating a condition that required selection of an unlikely combination of multiple viral mutations for the virus to escape immune control.

A control group immunized only with the LT adjuvant was not included in this study so we cannot rule out the possibility that the LT adjuvant alone may have stimulated non-specific responses that enhanced therapeutic efficacy. In mice, we showed LT has minimal impact on Th2 responses but substantially increases T cell proliferation and Th1 CD4+ T cell responses with a net outcome of shifting a Th1/Th2 response to primarily a Th1 response [26], [54]. Thus, LT, whether alone or as an adjuvant to the DNA vaccine, may improve therapy by increasing virus-specific or nonspecific Th1 antiviral cytokine responses or by activating latently-infected CD4+ T cells. The residual virus harbored by these cells would be more susceptible to clearance by the antiretroviral drugs or by local SIV-specific cytolytic CD8+ T cells induced by the DNA vaccine. In this setting, effective ART is likely important to preserve responsiveness to immunization and suppress re-infection of CD4+ T cells. Additional studies are needed to more precisely define the immune mechanisms of viral control afforded by the LT-adjuvanted DNA vaccine and to determine a possible therapeutic role of the LT adjuvant independent of the vaccine.

The number of animals employed in this study does not approach the size of efficacy trials in humans, a condition that raises caution about the interpretation of the outcome of macaque trials in general. For example, recent studies in macaques have shown a strong influence of polymorphic *Trim5α* alleles in SIV production [35], [55]. Other host factors, such as APOBEC and tetherin, also known to play a role in viral resistance [56], could influence virus burden. Polymorphic alleles for any one of these genes could work synergistically with antiretroviral drugs independent of the vaccine to suppress virus replication. Haplotyping of the animals used in this study clearly showed no association of a particular MHC class I or *Trim5α* allele with study outcome. However, we cannot rule out other intrinsic host factors that could influence the response to drugs and/or vaccines in SIV-infected macaques [57], [58], [59], [60].

In summary, our findings reiterate the importance of the gut in HIV infection and pathology [61], [62], [63] and provide the first evidence that vaccines capable of inducing mucosal responses may be able to afford a functional cure by targeting the persistent gut viral reservoir. The results also support the notion that an effective therapeutic vaccine could convert HIV-1 infected persons on highly active antiretroviral drug regimens into elite controllers, a term used to describe a subset of HIV-1 infected individuals who, like the macaques protected from viral rebound in this study, can maintain long-term suppression of viral load at very low levels in the absence of antiretroviral drugs. It is noteworthy that vaccine-induced control was achieved despite the use of a highly pathogenic primary SIV isolate that in infected macaques produces higher viremia, more rapid progression to AIDS, and was less effectively inhibited by the antiretroviral drugs used in this study than in HIV-infected patients undergoing HAART. The profound impact of vaccination in this setting suggests DNA vaccines may be able to induce a functional cure in HIV infected patients even if suboptimal drug regimens insufficiently control viral replication. Additional studies will be needed to determine the impact of this vaccine strategy in combination with more effective antiretroviral drug regimens and initiated at even later stages in the disease. These findings highlight the potential of a PMED DNA vaccine as an adjunct to drug therapy to improve HIV treatment and support further investigation of vaccines that target the mucosa, in general, as a means to reduce and perhaps even eradicate persistent viral reservoirs.

## Methods

### Ethics Statement and animal care

The University of Pittsburgh takes responsibility for humane care and use of laboratory animals in all research projects including those awarded by the Public Health Service. They are committed to comply with the Principles for Use of Animals, the National Institute of Health Guide for the Care and Use of Laboratory Animals, the Provisions of the Animal Welfare Act, and other applicable laws and regulations which are consistent with the recommendations of the Weatherall report “The Use of Non-human Primates in Research”. The University's Statement of Assurance is on file with the PHS, Office for Protection from Research Risks (A3187-01). The University of Pittsburgh is accredited by the American Association for the Accreditation of Laboratory Animal Care International (AAALAC). All experimental manipulations on rhesus macaques were approved by the University of Pittsburgh's Institutional Animal Care and Use Committee (protocol # 0710265). In addition, all studies were conducted after review by the GlaxoSmithKline (GSK) Institutional Animal Care and Use Committee and in accordance with the GSK Policy on the Care, Welfare and Treatment of Laboratory Animals. Animals were cared for by well established, competent clinical veterinary and animal caretaker staff. All animals received environmental enrichment throughout this study. All experimental procedures were performed under ketamine anesthesia and any discomfort or pain was alleviated by appropriate use of analgesic agents at the discretion of the attending veterinarian. Health parameters including CD4 T cell counts, weight, complete blood analysis, and evidence of opportunistic infection were monitored at least monthly. Early endpoints of disease were adopted and humane euthanasia was performed in accordance with guidelines as established by the 2007 American Veterinary Medical Association Guidelines on Euthanasia once manifestation of clinical AIDS or signs of fatal disease were noted.

### MHC class I and TRIM5 typing of rhesus macaques

Indian-origin rhesus macaques (*Macaca mulatta*) were MHC class I typed for currently known *Mamu* alleles (A*01, A*02, A*11, B*08, and B*17) by PCR sequence specific priming and direct sequencing as previously described [64]. Macaques were typed for *TRIM5* genotypes (TFP, Q, or CypA) that have been associated with either resistance or intermediate susceptibility to SIVmac251 [35] or SIVsmE660 [34] by PCR amplification of the C-terminal B30.2/SPRY domain of rhesus*TRIM5* utilizing purified genomic DNA and amplification protocols as described [34]. Resulting PCR fragments were cloned using the TOPO-TA vector system (*Invitrogen*), and *TRIM5* sequences for each animal were obtained from 4–6 clones. Genotypes were determined after alignment with *Geneious* software and classified as described [34], [55].

### Viral challenge and disease progression

Macaques were challenged intravenously with 1 ml of RPMI containing 100 TCID_50_ of cryopreserved SIV/DeltaB670 and monitored for clinical signs of AIDS as described [27]. CD4^+^ T cell counts were determined by flow cytometry as described [60].

### Anti-retroviral therapy (ART)

Macaques received daily subcutaneous injections of 20 mg/kg (R)-9-(2-phosphonomethoxypropyl) adenine (PMPA; Gilead Sciences, Foster City, CA) and twice daily oral administrations of 16 mg lopinavir/ritonavir (Kaletra®; Abbot Laboratories, Abbott Park, Illinois) (approximately 13 mg/Kg).

### Collection of gut associated tissues

A 15–20 cm section of jejunum and axillary and mesenteric lymph nodes were surgically removed from each macaque at time-points indicated in Figure 1B as described [27]. All animals recovered uneventfully from surgery without post-surgical diarrhea and weight loss.

### Mononuclear cell (MNC) isolation from jejunal resections and lymph nodes

Mononuclear cells were isolated from fresh lymph nodes and jejunum (lamina propia lymphocytes or LPL) using a well-established enzymatic extraction protocol as previously described [22], [27]. This procedure yields at least 20–80×10^6^ cells, a sufficient number of cells for analysis of both T cell function and specificity.

### SIV DNA vaccines

Codon-optimized cDNA of SIV/17E-Fr (Genbank #AY033146) *RT* (nucleotides 2852–3117), *Nef* (nucleotides 9175–9868), *Gag p17/27* (nucleotides1053–2142) and *gp120* (nucleotides 6604–8895) were used as templates to construct the vaccine. To reduce potential immunomodulatory effects, RT was inactivated by mutating W229 to K (W229K), and *Nef by* removal of the first 97 amino acids. Contiguous *RT*-*Nef-Gag* and gp120 sequences were inserted into the vector cassette, p7313, containing IE CMV promoter/intronA, a *Not I*/*BamHI* multiple cloning site, the bovine growth hormone poly-adenylation signal, and bacterial maintenance sequences (GSK, Stevenage, U.K.) to allow expression of a fusion protein antigen (pSIV-RNG) and *gp120* antigen (pSIV-gp120), respectively (Fig. 1A).

### Heat-labile enterotoxin (LT) genetic adjuvant

The plasmid expressing A and B subunits of the *Escherichia coli* heat-labile enterotoxin (LT) was previously constructed and tested for expression as described [26].

### Particle-mediated DNA immunization

The pSIV-RNG and pSIV-gp120 plasmids were coated onto separate gold particles as described [65], and mixed prior to administration. Macaques were immunized with 32 µg (16 µg pSIV-RNG and 16 µg pSIV-gp120) co-delivered with or without 3.2 µg of LT into 16 target sites using the PowderJect® XR1 gene delivery device (PowderJect Vaccines, Inc., Middleton, WI) as described [66].

### Analysis of viral loads in plasma and tissues

Virion-associated RNA in plasma and tissues was quantified by real time RT-PCR using primers specific for the SIV long terminal repeat (LTR) as described [22], [32]. Cell-associated SIV transcripts were quantified in Trizol extracted RNA from tissues or purified mononuclear cells. 250 ng of purified RNA was analyzed by real-time RT-PCR using forward and reverse primers:probe specific for the U5 region of 5′LTR and the primer binding site. Because these primers amplify a 92 bp fragment found only in SIV transcripts prepared for packaging into virions, they can be considered a reliable indicator of virus production in the tissues. Forward primer: U5PBSF - GAA ACC GAA GCA GGA AAA TC; reverse primer: U5PBSR- CTG CCT TCA CTC AGC CGT ACT, probe: U5PROBE- 6FAM AGG AGT CTC TGA CTC TCC TTC AAG TCC CTG TT TAMARA. The assay has an amplification efficiency of 97% and a sensitivity of 3 copies/reaction.

### IFN-γ ELISpot assay

IFN-γ ELISpot assay was performed following stimulation of purified mononuclear cells with pools of 15mer peptides overlapping by 11 amino acids spanning SIV Gag, RT, Nef, and Env as previously described [8], [32]. Responses significantly higher than background levels (twice the number of IFN-γ spot forming cells or SFC from untreated PBMC plus 10 spots) were considered positive.

### Intracellular cytokine staining assay

Multiparameter intracellular cytokine staining (ICS) assays were performed on mononuclear cells stimulated with peptide pools spanning SIV *gag* and *env* as previously as described [42], [67]. Cells were stained with the following antibodies (BD Biosciences, San Jose, CA): CD3-Pacific Blue™ (rhesus, cloneSP34-2); CD8-APC-Cy7 (rhesus, clone RPA-T8), CD4-PerCP (rhesus, clone L200), FITC-CD107a/b (human clones H4A3, H4B4), PE-IL-2 (human, clone MQ1-17H12), APC-TNFα (rhesus, clone MAb11), and PE-Cy7-IFN-γ (human, clone 4S.B3) and analyzed using an LSR II flow cytometer (BD Biosciences) and FlowJo software (Tree Star, Inc., Ashland, OR). Events were subjected to a lymphocyte gate by a forward scatter versus SSC. Live T cells were identified by a live/dead versus CD3+ plot. Following identification of CD8+ and CD4+ T cells, a gate was made for each function and a Boolean gate platform was used to create all possible combinations. All data are reported after subtraction of background staining of unstimulated cells obtained at the same timepoint. Background levels from unstimulated cells or cells stimulated with irrelevant peptide (HBsAg) typically ranged from 0.04–0.18% for single effector function analysis and 0.00–0.03% for multiple (2–4) functions. Baseline single function responses (week 0, prior to infection) in each macaque ranged from −0.06–0.04%.

### Analysis of antibody responses

SIV-gp120 specific IgG binding antibody titers were measured by enzyme-linked immunosorbance assay (ELISA) as described using recombinant SIVmac239 gp120 for capture antigen (Intracel, Issaquah, WA) and a rabbit anti-IgG (heavy and light chains conjugated to alkaline phosphatase (Accurate Chemical, Westbury, NY) to detect antibody bound to the capture antigen [22]. Viral neutralization titers were determined as described [68]. Briefly, serial dilutions of plasma from vaccinated animals were incubated with 200 TCID_50_ of the SIV isolates 17E-Fr or Delta-B670 and incubated for 1 hour before addition to Tzm-bl cells. After 48 hours, the levels of infection were determined using Bright-Glo reagents (Promega, Madison, WI) and measurement of relative light units. The neutralizing titer was identified as the reciprocal of the highest plasma dilution able to inhibit virus infection by 70% as compared to virus controls. Neutralization titers were confirmed in duplicate assays.

### Statistical analyses

Statistical comparisons of protection from viral rebound, viral loads in tissues and blood, and mucosal and systemic immune responses were performed between each vaccine group and the controls using GraphPad Prism statistical software. The primary endpoints for comparing therapeutic efficacy between vaccinated animals and controls were protection from viral rebound, as defined by containment of viral loads in plasma <100 viral RNA copies/ml for a period of at least 5 months (21 weeks) after stopping ART and protection from progression to AIDS as defined by maintenance of CD4 counts above 50% of the baseline pre-infection levels for the duration of the study. Differences in protection from viral rebound and/or CD4 decline between groups were determined by one-sided Fisher's exact test. Differences in serial measurements of viral RNA loads in plasma or T cell responses between each vaccine group and controls were determined by two-sided Wilcoxon rank-sum test and repeat measures ANOVA, respectively. Differences between mean viral RNA loads in gut and lymph node tissues or T cell responses at individual time points were determined by two-sided Mann-Whitney to compare 2 groups or one-way ANOVA and Kruskal-Wallis post-test to compare 3 groups. Correlations between virus loads in tissues and plasma or between immune responses and virus loads in tissues or plasma were determined by Spearman's rank correlation test. A significance threshold of 0.05 was used for each statistical test and, where appropriate, adjustments for multiple comparisons were made.
